# Frequency-Enhanced and Multi-Scale Feature Fusion YOLOv11 for Low-Illumination Weak Projectile Target Recognition in Linear Array CCD Sensor

**DOI:** 10.3390/s26175346

**Published:** 2026-08-24

**Authors:** Haorui Han, Hanshan Li, Keding Yan

**Affiliations:** School of Electronic and Information Engineering, Xi’an Technological University, Xi’an 710021, China; hanhaorui@st.xatu.edu.cn (H.H.); lihanshan269@163.com (H.L.)

**Keywords:** YOLOv11, low illumination, false targets, small target detection

## Abstract

To solve the problem where the low contrast and extremely small target size in the three-sky-screen target-integrated linear array CCD sensor measurement system under low-illumination conditions make it difficult to accurately identify projectile targets, this paper proposes a method of Frequency-Enhanced and Multi-scale Feature Fusion YOLOv11 (FEMFF-YOLOv11). It introduces a frequency-domain enhancement module in the backbone to improve feature discriminability, and deformable offset convolution is incorporated to handle geometric deformations. It also adds a multi-scale attention aggregation module in the neck to strengthen weak target features and suppress false targets such as near-lens flying objects. The detection head is optimized by replacing the low-resolution P5 layer with a high-resolution P2 layer for better projectile target localization. Experiments are conducted on a self-built linear array CCD projectile dataset. The results demonstrate that compared with YOLOv11 and other mainstream algorithms, our method achieves 87.35% precision and 85.13% recall under 300 lx low-illumination conditions. It also maintains 85.91% precision and 82.56% recall even at 50 lx, significantly outperforming all competitors.

## 1. Introduction

**Figure 1:**
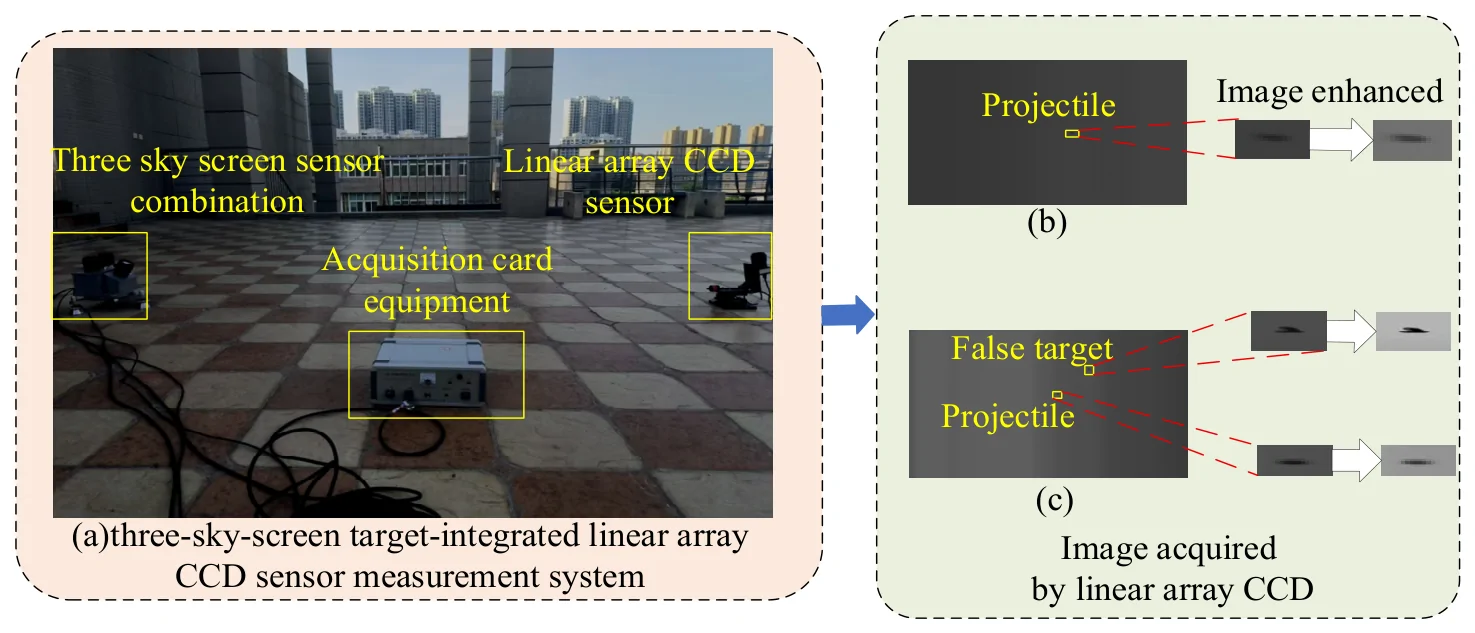
Three-sky-screen target-integrated linear array CCD sensor measurement system: test configuration and captured images. (**a**) three-sky-screen target-integrated linear array CCD sensor measurement system; (**b**) Images of projectiles collected under low-illumination conditions; (**c**) Images of projectile with false target collected under low-illumination conditions.

The precise measurement of projectile flight parameters is a key technical support for the performance evaluation and motion analysis of automatic-fire weapons [1,2]. The light screen sensor testing system can calculate flight parameters by precisely capturing the instants when the projectile crosses each light screen sensor and combining them with spatial geometric relationships [3,4]. Among them, the measurement system that integrates three sky-screen target sensors and a linear array CCD sensor [5,6] possesses high integration and high sensitivity, enabling multi-parameter measurements in both single-shot and continuous-fire modes, and offers irreplaceable performance advantages over traditional systems in parameter testing of automatic-fire weapons and motion analysis of static blast fragmentation groups [7,8]. However, the system is deployed in an outdoor environment with the sky as the background light source, and its performance is significantly affected by environmental light [9,10]. Specifically, the sky-screen target sensors acquire analog signals. Although the signal-to-noise ratio decreases under low-illumination conditions, the instant of the projectile crossing the sky-screen target can still be effectively extracted through gain adjustment, threshold optimization, filtering and denoising [11]. In contrast, the linear array CCD, as a spatially resolved imaging sensor, relies on accurate identification of the pixel row where the projected light spot is located in the image to precisely extract the moment of passage. Under low-illumination conditions, the contrast between the projectile and the background is severely reduced, and the target pixel row is submerged in background noise. This not only leads to a sharp drop in the extraction accuracy of the detection moment, but also directly affects the measurement reliability of key flight parameters such as the projectile’s speed and flight direction [12,13]. Taking the test setup shown in Figure 1a as an example, it is evident that the imaging problem of the projectile on the linear array CCD sensor under low illumination is particularly prominent.

When the test equipment is deployed in an outdoor low-illumination environment, the background luminous flux is severely insufficient [14,15]. At this time, the imaging contrast of the linear array CCD sensor decreases significantly, the grayscale difference between the target and the background is reduced, and the projectile’s edges tend to become blurred, which seriously restricts the effective recognition of the projectile target (as shown in Figure 1b) [16,17]. In addition, when the projectile crosses the measurement light screen at a high speed, its effective imaging pixels on the linear array CCD sensor are fewer than eight, making it a typical weak and small target [18]. The available spatial shape features are extremely limited, and its geometric information is difficult to be effectively characterized under low-illumination imaging. Moreover, in outdoor scenes, near-lens flying objects (such as mosquitoes, sand grains, and dust) share certain similarities with the real projectile in terms of the imaging scale, and are therefore easily misdetected as projectile targets, causing severe false-target interference and further increasing the difficulty of recognition (as shown in Figure 1c) [19,20]. Facing the above complex imaging conditions with multiple sources of interference, traditional image enhancement algorithms (such as histogram equalization [21] and filtering [22]) are difficult to use to effectively preserve the edge details of weak and small targets while suppressing background noise, and conventional target detection networks lack adaptive mechanisms for low-illumination conditions and are prone to misdetecting false targets as real projectiles, leading to low recognition accuracy. Therefore, how to effectively enhance the features of weak and small projectile targets and achieve accurate recognition under low-illumination conditions is a critical problem that urgently needs to be solved in linear array CCD projectile target recognition.

To address the challenges of low contrast between the projectile and background, weak and small features, and interference from near-lens false targets under low-illumination conditions in the three sky-screen target-integrated linear array CCD sensor projectile measurement system, this paper proposes a low-illumination weak and small projectile target recognition network named FEMFF-YOLOv11. The network takes frequency-domain feature enhancement and content-guided multi-scale feature aggregation as its core components, supplemented by deformable convolution and improved detection layers to enhance the network’s geometric adaptation capability and target localization ability. The proposed method effectively improves the projectile recognition rate and measurement reliability of the system in complex outdoor low-illumination environments, and provides reliable technical support for parameter testing and analysis of automatic-fire weapons under low-illumination operating conditions. The main innovations and contributions of this paper are as follows:

1. An Adaptive Wavelet Transform Feature Enhancement (AWTFE) module is embedded in the backbone network. This module performs wavelet-domain decomposition on low-illumination images to extract low-frequency approximation components and high-frequency detail components. It then selectively enhances the high-frequency details in the frequency domain, effectively suppressing background noise while highlighting weak high-frequency information such as projectile edges. This addresses the issues of low contrast between targets and the background and the difficulty in extracting edge features under low-illumination conditions, significantly improving the network’s feature representation capability for low signal-to-noise-ratio images.
2. A Deformable Offset Convolution (DOC) module is introduced into the backbone network. By using its dynamic sampling point offset mechanism, the convolution kernel can adaptively adjust the sampling positions according to the actual geometric shape of the projectile, effectively fitting the irregular geometric shape of the projectile. This addresses the problem in feature extraction caused by target deformation and enhances the network’s perception capability for projectiles with different spatial shapes.
3. A Content-guided Multi-scale Feature Aggregation (CMFA) module is designed and added into the neck network. Based on a content-guided attention mechanism, it first fuses channel and spatial attention to generate a coarse-grained spatial attention map, which is then refined under the guidance of input feature content to produce channel-specific detail slices. To address insufficient local feature modeling in large-scale feature maps, a split-wise processing strategy is incorporated to refine global fusion into local regions. Through a “channel–spatial–pixel” three-level collaborative attention mechanism, the module progressively focuses on projectile edge features from coarse to fine, ensuring that critical local details are not overlooked. This enables precise pixel-level discrimination between projectiles and near-lens false targets, effectively suppressing complex false-target interference.
4. To address the loss of spatial information in weak and small projectile targets after multiple down-sampling operations, the original low-resolution P5 detection layer in YOLOv11 is removed and a high-resolution P2 feature layer is introduced, further strengthening the network’s capability for detail perception and localization of weak and small targets.
5. The above four improvement modules synergistically operate from four dimensions: frequency-domain feature enhancement (AWTFE), spatial geometric adaptation (DOC), false-target suppression (CMFA), and feature spatial resolution preservation (P2 layer). Together, they form a comprehensive processing pipeline that significantly improves the recognition accuracy and robustness of the linear array CCD sensor for weak and small projectile targets under low-illumination conditions.

## 2. Related Work

Under low-illumination conditions, in images acquired by the linear array CCD sensor, the low contrast of projectile targets, blurred edges, and interference from near-lens flying objects pose severe constraints on the three-sky-screen target-integrated linear array CCD measurement system. To overcome these problems, researchers have conducted extensive research on target recognition for linear array CCD images. Existing approaches mainly fall into two categories: one is traditional digital image processing methods that rely on hand-crafted features and fixed algorithmic procedures [23]; the other is deep learning methods [24] that automatically learn hierarchical feature representations through data-driven approaches. The current research status of the two types of methods is analyzed separately below.

In terms of traditional digital image processing methods, they mainly rely on hand-crafted features and fixed algorithms. For example, Huang [25] utilized an FPGA + DSP collaborative processing architecture to achieve sub-pixel edge detection of linear array CCD images through steps such as image smoothing filtering, gradient operator-based edge coarse localization, fitting interval search, and least-squares straight-line fitting for precise localization. Chen [26] combined the two-dimensional Otsu algorithm with gradient ellipse fitting to solve the problems of low precision and poor stability in dynamic attitude detection of linear array CCDs, by adopting adaptive threshold segmentation in the coarse detection stage and gradient ellipse fitting sub-pixel localization in the fine detection stage. The above methods can effectively extract target edge information under conventional imaging conditions and achieve satisfactory detection performance. However, such methods are heavily dependent on manually designed features and fixed rules. Under conditions where the grayscale difference between the target and background is small, their detection accuracy and anti-interference capability are insufficient [27,28].

In contrast, deep learning methods demonstrate stronger robustness and adaptability in linear array CCD target recognition by automatically learning multi-level feature representations. For example, Wu [29] used a linear array CCD camera equipped with active light sources to capture images, applied an improved Retinex image enhancement algorithm with multi-scale decomposition to improve image contrast, and then built an improved VGG19 network model for efficient target segmentation. Tao [30] addressed the problems of low manual detection efficiency and high misdetection and false-detection rates in surface micro-damage inspection of products, by adopting a two-stage strategy that combines linear array CCD image acquisition with YOLOv3 for coarse localization and a level-set algorithm for fine localization, achieving rapid detection of micro-damage. The above methods avoid the limitations of hand-crafted features through a data-driven manner and can effectively cope with complex imaging interference [31]. However, the scenes they address are mostly industrial surface inspection and crack recognition, which differ from the characteristics of linear array CCD imaging under low illumination, where projectile targets are weak and small and difficult to distinguish from false targets.

In projectile target detection, some researchers have conducted preliminary explorations based on cameras and conventional illumination conditions. Hu [32] proposed an improved YOLOX algorithm, which enhances small-target detection capability by introducing attention mechanisms and improved loss functions. Ji [33] proposed an improved SSD method that optimizes the feature extraction network structure. He [34] proposed the YOLOv5-FD method, which improves small-sample projectile detection accuracy by adding micro-target detection layers, introducing coordinate attention, and applying MAML meta-learning. The above studies have laid an important foundation for projectile target detection, but they are all based on array imaging and conventional illumination conditions.

Based on the analysis of existing studies, linear array CCD-based low-illumination weak and small projectile target recognition still faces the following key challenges:

1. Under low-illumination conditions, the contrast between the projectile target and the background decreases significantly, and edge and shape information is submerged in noise. Existing methods lack specialized enhancement mechanisms for low-illumination degraded features, making it difficult to effectively separate the target from the background.
2. In outdoor scenes, near-lens flying objects such as mosquitoes and dust share certain similarities with real projectiles in terms of imaging scale and appearance features on the linear array CCD sensor. Moreover, under low-illumination conditions, their edges are also blurred. Existing methods lack effective mechanisms for distinguishing true targets from false ones, leading to a significant increase in false alarm rates.
3. The projectile target occupies an extremely low number of pixels in the linear array CCD image and exhibits irregular shapes. Existing projectile detection methods all adopt conventional convolutional network architectures and do not perform adaptive sampling point modeling for the geometric deformation of such irregular targets, resulting in insufficient feature extraction and unstable target representation.

To address the above problems, this paper proposes a linear array CCD low-illumination weak and small projectile target recognition method based on frequency-domain enhancement and multi-scale feature fusion, named FEMFF-YOLOv11. The network first enhances the edge responses of all candidate targets through a frequency-domain enhancement module. On this basis, it adopts a deformable offset convolution module to extract the geometric features of irregular projectiles. Finally, a multi-scale feature fusion mechanism is incorporated to discriminate between projectiles and false targets, thereby achieving accurate recognition.

## 3. Projectile Recognition Method for Low-Illumination Linear Array CCD Images Based on Frequency Enhancement and Multi-Scale Feature Fusion

### 3.1. Challenges in Low-Illumination Projectile Target Recognition for Linear Array CCD and the Design of FEMFF-YOLOv11 Network

**Figure 2:**
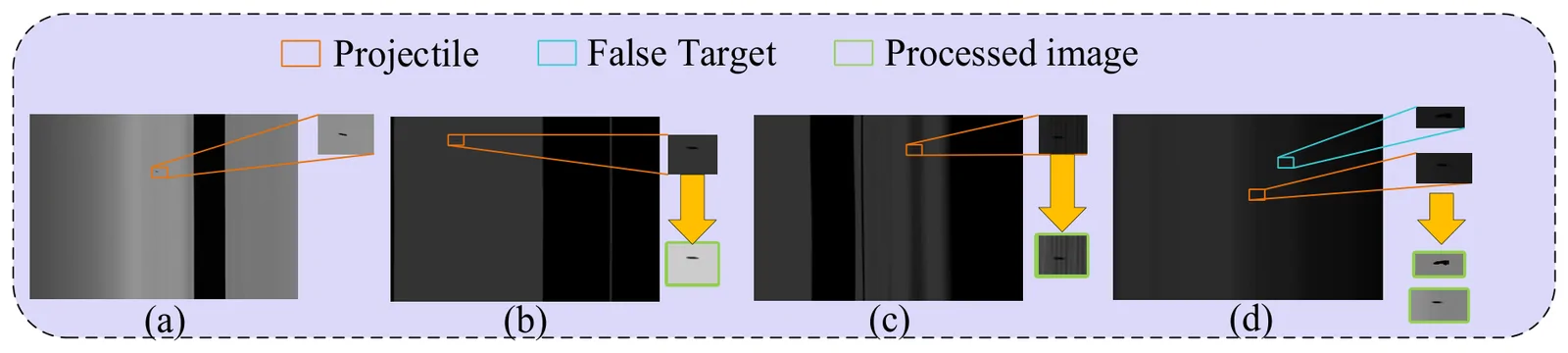
Linear array CCD projectile images under different scenes. (**a**) Images of projectiles captured under low-illumination conditions; (**b**) Images of projectiles captured under medium-low-illumination without interference; (**c**) Images of projectiles captured under medium-low-illumination with background submerged; (**d**) Images of projectiles captured under extreme-low-illumination with false targets.

In the projectile parameter testing system integrating three sky screens and a linear array CCD sensor, an industrial-grade black-and-white CCD microscopic camera is employed, with a pixel size of 10 μm × 10 μm and a maximum line frequency of 50 kHz. The system is deployed outdoors, utilizing sky light as the passive illumination source. Figure 2 and Table 1 shows a typical projectile target image captured by the linear array CCD under low-illumination conditions, along with the existing problems.

Based on the analysis of image characteristics and recognition challenges in the four scenes above, three core difficulties exist in low-illumination weak and small projectile target recognition for linear array CCD:

1. As shown in Figure 2a, the projectile occupies fewer than eight effective imaging pixels, resulting in extremely limited spatial shape features. The linear array CCD sensor adopts a one-dimensional line-by-line scanning mechanism. When the projectile passes through the measurement light screen at a high speed, it covers only a very small number of pixel units, and its geometric contour is severely compressed under low-resolution imaging. Conventional target detection networks rely on rich spatial texture information for feature extraction, but in this scene, the available discriminative information is extremely scarce, making it difficult for the model to establish a stable target representation. In addition, the projectile often exhibits irregular shapes in linear array CCD images, which further increases the difficulty of feature extraction. As a result, traditional convolution operations cannot effectively fit the actual spatial distribution of the target, and the feature response intensity is greatly weakened.
2. As shown in Figure 2b,c, the decrease in contrast causes target edges to be submerged in noise. From the given images, it is already difficult to distinguish the projectile target with the eye, and overlapping background textures further aggravate target confusion. Specifically, under low-illumination conditions, the background luminous flux is severely insufficient, which compresses the grayscale dynamic range of the linear array CCD output image. The originally clear projectile edges degenerate into blurred grayscale transitions due to the sharp drop in contrast. When the projectile overlaps with cluttered background textures such as branches in spatial position, their grayscale distributions become highly similar, and the target contour information is completely submerged in background noise. At this point, not only is it difficult for the human eye to distinguish the target, but the edge detection operators of existing image processing algorithms also fail to respond effectively.
3. As shown in Figure 2d, extreme-low illumination further compresses the differences between projectiles and flying objects in terms of shape regularity, edge sharpness, and grayscale distribution, reducing inter-class discriminability. Under low-illumination conditions, projectiles and near-lens flying objects (such as mosquitoes, sand grains, and dust) exhibit relatively obvious differences in shape regularity, edge clarity, and grayscale uniformity. However, when the illumination drops to the 50 lx level, the inter-class discriminative features between projectiles and false targets are significantly compressed. This makes it difficult for the classification network to construct an effective decision boundary in the feature space, and false targets are easily misdetected as real projectiles, leading to a notable increase in the false alarm rate.

**Figure 3:**
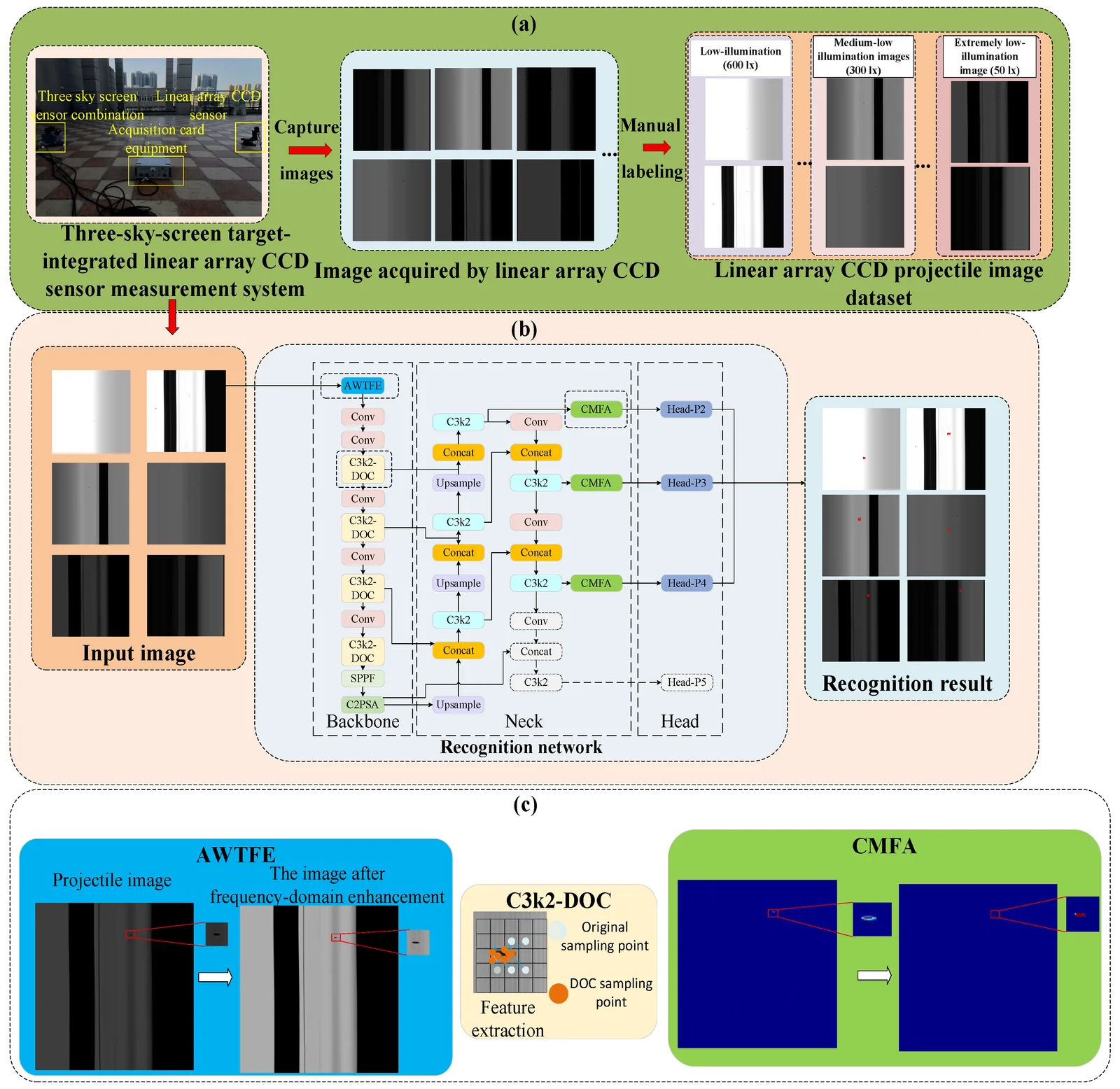
Overall processing flow chart of the linear array CCD low-illumination weak and small projectile target recognition method based on FEMFF-YOLOv11. (**a**) Dataset collection and construction; (**b**) FEMFF-YOLOv11 recognition process; (**c**) Processing procedure.

The above problems together constitute the difficulties in linear array CCD low-illumination weak and small projectile target recognition, corresponding, respectively, to three aspects: sparse target features, degradation of the imaging environment, and external scene interference. To solve these problems, this paper proposes a low-illumination weak and small projectile target recognition method for linear array CCD based on frequency-domain enhancement and multi-scale feature fusion, named FEMFF-YOLOv11. The method uses frequency-domain feature enhancement to restore weakened edge details in low-illumination images. It adopts a deformable offset convolution module to fit the irregular shapes of projectiles. It also designs a content-guided multi-scale feature aggregation module to precisely distinguish projectiles from false targets at the pixel-level feature space. By combining these strategies, the method achieves reliable projectile recognition in complex low-illumination scenes. The overall processing flow of the method is shown in Figure 3.

The overall processing flow of the method consists of two stages, with the intermediate outputs of each stage and the effect of each module illustrated in Figure 3. In the first stage, a multi-light-screen fusion detection system composed of sky-screen targets and a linear array CCD sensor is used to capture projectile target images under different illumination conditions. After preprocessing and manual annotation, a linear array CCD projectile image dataset is constructed to provide a data foundation for network training and performance evaluation. The image transformation from raw capture to annotated input is shown in the Figure 3a. In the second stage, the preprocessed images are fed into the FEMFF-YOLOv11 network, as shown in the Figure 3b, which performs forward inference sequentially through the backbone network, neck network, and detection head, ultimately outputting the recognition results and position information of the projectile targets. In Figure 3c, the influence effects of each module on the image are visualized through corresponding feature maps, including AWTFE, DOC, and CMFA.

FEMFF-YOLOv11 is built upon the YOLOv11 architecture and incorporates three targeted improvements to address the three core difficulties mentioned above:

1. Frequency-domain enhancement: An Adaptive Wavelet Transform Feature Enhancement module is embedded in the backbone network. Through wavelet-domain decomposition and selective enhancement of high-frequency details, this module suppresses background noise. At the same time, it highlights weak high-frequency information such as projectile edges. In this way, it improves the discriminability of low-illumination targets.
2. Geometric adaptation: A deformable offset convolution module is introduced into the backbone network. By using its dynamic sampling point offset mechanism, the convolution kernel adaptively adjusts the sampling positions according to the actual shape of the projectile. This effectively fits the irregular shapes of the projectile and enhances the network’s perception of geometric features of weak and small targets.
3. False-target discrimination: A content-guided multi-scale feature aggregation module is designed in the neck network. It employs a “channel-space-pixel” three-level collaborative attention mechanism. Through this mechanism, the module progressively focuses on the key regions of the projectile from coarse to fine. As a result, it achieves precise discrimination between projectiles and near-lens flying objects at the pixel-level feature space and effectively suppresses complex false-target interference.

In addition, to address the problem of spatial information loss of weak and small projectile targets after multiple down-sampling operations, the low-resolution P5 detection layer in the original YOLOv11 network is removed and a high-resolution P2 feature layer is introduced, further strengthening the network’s detail perception and localization accuracy for weak and small targets.

### 3.2. Adaptive Wavelet Transform Feature Enhancement Module for Low-Illumination Images

**Figure 4:**
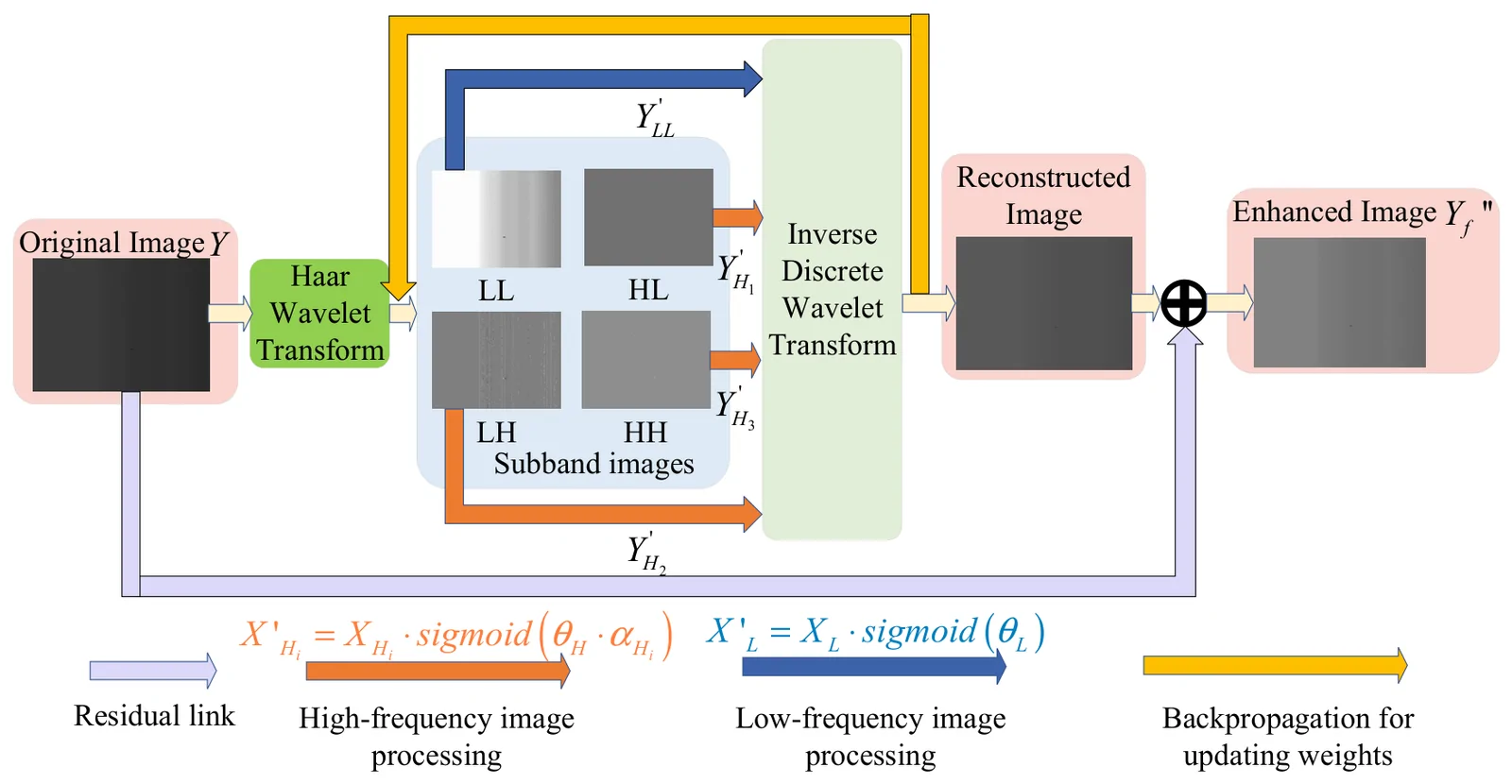
Adaptive Wavelet Transform Feature Enhancement module for low-illumination linear array CCD images.

Under low-illumination conditions, the grayscale values of the projectile target and background become similar, significantly reducing contrast. The target’s edge and shape information is severely degraded, blurring its contour and making effective separation from the background difficult. The original YOLOv11 network struggles to cope with the situation where target signals are overwhelmed by background noise under low illumination. To address this issue, this study introduces an Adaptive Wavelet Transform Feature Enhancement (AWTFE) module into the backbone network of YOLOv11. This module first decomposes the input low-illumination linear array CCD image through discrete wavelet transform, producing different frequency band components. In this way, the projectile target is preliminarily separated from the background in the frequency domain. Subsequently, the module adaptively adjusts each frequency band component using learnable frequency-domain weights. Through this adjustment, low-frequency background energy is suppressed, while high-frequency components that predominantly contain the target information are selectively amplified. Finally, the enhanced frequency-domain features are reconstructed into an enhanced image through inverse discrete wavelet transform. In this reconstructed image, the projectile target, which was originally submerged in the low-illumination image, is effectively recovered and highlighted. This provides clearer feature input for subsequent target localization and recognition. The processing flow of the AWTFE is illustrated in Figure 4.

Let the input low-illumination feature map be denoted as $Y\in R^{B\times C\times H\times W}$, where B is the batch size, C is the number of channels, and H and W are the height and width of the feature map.

To fully use frequency-domain information for target signal enhancement and background noise suppression, the AWTFE module first performs a one-level discrete wavelet transform (DWT) on the input feature map. This operation yields four sub-band images. Among these, LL represents the low-frequency approximation coefficients, which mainly characterize the global structure and dominant background information of the image. They correspond to the large-area smooth grayscale regions that are typically seen in low-illumination images. The coefficients LH, HL, and HH are the high-frequency detail coefficients, containing edge and texture information in the horizontal, vertical, and diagonal directions. The weak edges, contours, and boundary features of the slender projectile are predominantly concentrated in these high-frequency components. Through the wavelet transform, the projectile target and the low-illumination background are preliminarily separated in the frequency domain.

Considering that the low-frequency coefficients determine the overall brightness and background distribution of the image, while the high-frequency coefficients carry the edge details of the projectile target, this paper designs an adaptive frequency-domain weight enhancement mechanism. It achieves a compromise between background suppression and structure preservation for the low-frequency components, and implements direction-aware selective edge enhancement for the high-frequency components.

For the low-frequency coefficient LL, which determines the overall brightness and background distribution of the image and is susceptible to large-area noise contamination under low-illumination conditions, a low-frequency enhancement weight is defined to suppress background interference while preserving necessary structural information as follows:

(1) $$W_{LL}=Sigmoid\left(P_{LL}\right),$$

where $P_{LL}$ is a learnable parameter with an initial value of −1 ($W_{LL}\approx 0.27$), enabling the model to preferentially suppress low-frequency background components at the initial stage, thereby preventing large-area background information from masking the weak high-frequency details of the projectile. As training proceeds, the model can adaptively adjust the degree of low-frequency information retention based on the loss feedback.

In linear array CCD imaging, projectiles appear slender and inclined, resulting in significant differences in the distribution of their high-frequency edge energy along the horizontal, vertical, and diagonal directions. Considering this, a direction-aware high-frequency enhancement strategy is adopted in this paper to process the high-frequency coefficients in a differentiated manner. The direction-aware high-frequency weights are defined as follows:

(2) $$W_{H_{i}}=Sigmoid\left(P_{H}\cdot P_{H_{i}}\right),i\in \left\{1,2,3\right\},$$

Here, $P_{H}$ denotes the global high-frequency weight, which controls the overall high-frequency enhancement intensity; $P_{H_{1}}$, $P_{H_{2}}$, and $P_{H_{3}}$ are the dedicated weights corresponding to the horizontal (HL), vertical (LH), and diagonal (HH) directional sub-bands, respectively, forming a direction-aware enhancement mechanism. This design enables the model to selectively enhance the primary edge directions of the projectile while suppressing noise responses in irrelevant directions. The enhanced frequency-domain coefficients are computed as follows:

(3) $${Y_{LL}}^{\prime }=Y_{LL}\cdot W_{LL},$$

(4) $${Y_{H_{i}}}^{\prime }=Y_{H_{i}}\cdot W_{H_{i}},$$

Here, ${Y_{LL}}^{\prime }$ denotes the enhanced low-frequency coefficients, and ${Y_{H_{i}}}^{\prime }$ denotes the enhanced high-frequency coefficients.

The four enhanced frequency-domain coefficients are then reconstructed into a spatial-domain feature map $Y^{\prime \prime }$ through the Inverse Discrete Wavelet Transform (IDWT). Under the effect of frequency-domain enhancement, the projectile target signals that were originally weakened by low illumination are effectively compensated. Background interference is significantly suppressed, and the contrast between the target region and the background is restored. This provides a high signal-to-noise-ratio input feature map for the subsequent backbone network.

To further stabilize the gradient flow during training and preserve the effective information in the original input features, this study introduces a residual connection mechanism that adaptively fuses the original feature map with the enhanced feature map. The final output of the AWTFE module is given as follows:

(5) $${Y_{f}}^{\prime \prime }=Y+\alpha \cdot Y^{\prime \prime },$$

where $\alpha =Sigmoid\left(P_{\alpha }\right)$ is a learnable residual weight. This mechanism enables the network to adaptively adjust the contribution of the wavelet-enhanced features based on the loss of feedback during training.

Through the above processing, the AWTFE module achieves selective amplification of projectile target signals and effective suppression of background noise in the frequency domain, providing a high signal-to-noise-ratio input feature map for the subsequent backbone network.

### 3.3. Adaptive Feature Extraction for Geometric Deformation of Projectile Based on Deformable Offset Convolution

**Figure 5:**
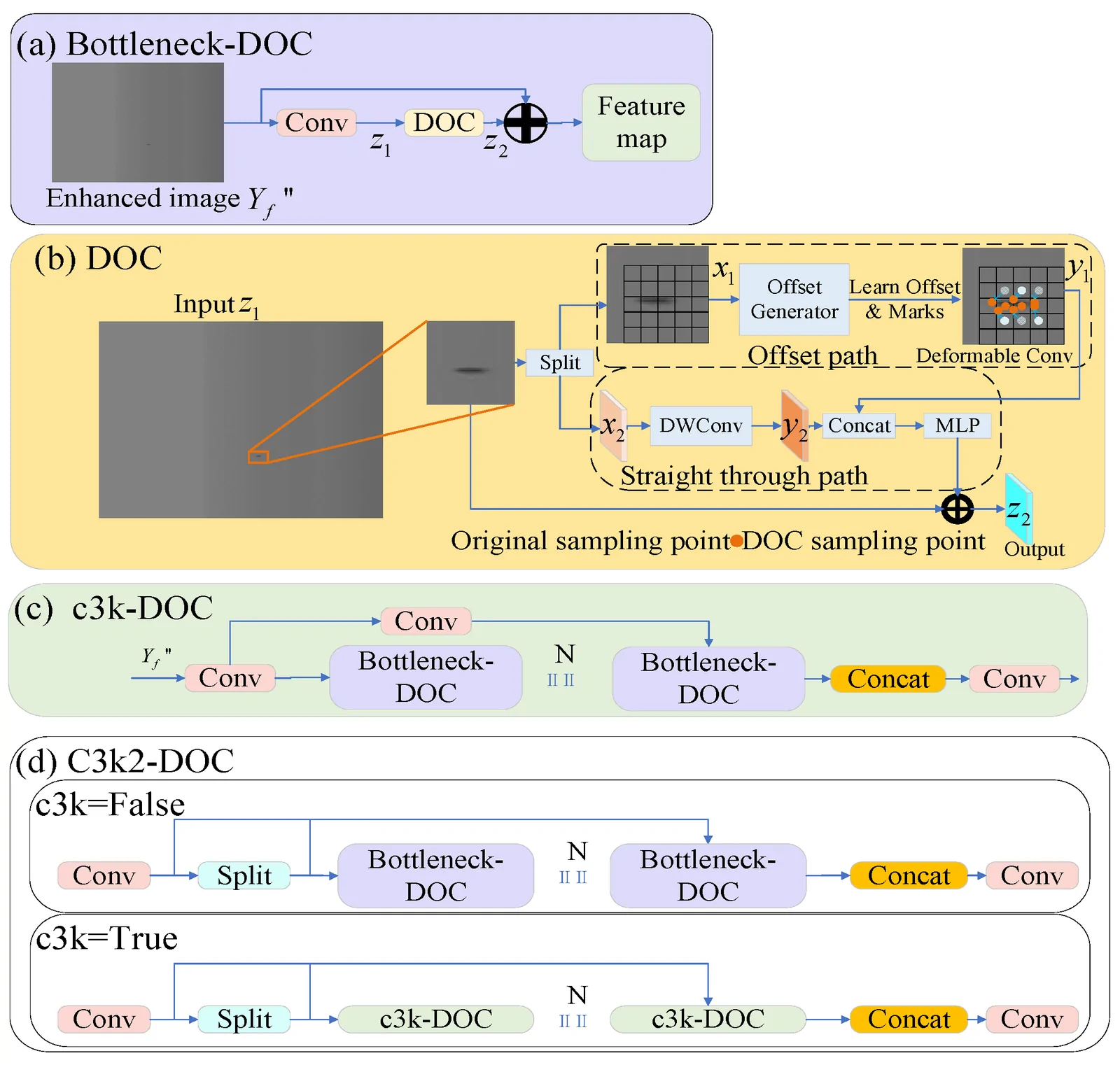
C3k2-DOC structure diagram.

After frequency-domain enhancement by the AWTFE module, the contrast of low-illumination projectile images is significantly improved, providing a high-contrast input feature map for the backbone network. However, projectiles exhibit irregular shapes in linear array CCD images, making it difficult for the fixed sampling grid of the Bottleneck module in the YOLOv11 backbone to effectively extract their deformation features. The Deformable Convolution Network (DCN) enables convolution kernels to adaptively adjust sampling positions based on target shapes through learnable sampling point offsets. Due to the extremely low pixel occupancy of projectiles and the weak and irregularly distributed contour information after deformation, the DCN fails to stably converge to accurate positions. To address this issue, this study proposes the Bottleneck-DOC module based on the DCN, which enhances the adaptability of convolution kernels to deformed projectile morphologies by optimizing the offset learning strategy. Its overall structure is shown in Figure 5a. To further strengthen the network’s feature extraction capability for deformed projectile morphologies, this study replaces the standard Bottleneck in the C3K module with Bottleneck-DOC, forming the C3K-DOC module (Figure 5c). This enables each Bottleneck unit to adaptively adjust sampling positions through the dynamic sampling point offset mechanism to fit the actual projectile morphology, thereby enhancing contour perception. Furthermore, it is embedded into the C3K2 module to form the C3K2-DOC module (Figure 5d), with the C3K mode enabled. The multi-scale convolution kernels are utilized to expand the receptive field and reinforce local texture and contour representation, thereby enhancing the network’s feature extraction capability for deformed projectile morphologies.

The DOC module proposed in this paper is shown in Figure 5b. Based on the standard DCN offset learning, this module introduces a dual-path design. Specifically, the DOC unit first splits the input feature map along the channel dimension into two feature maps, $x_{1}$ and $x_{2}$, through the Split operation. Subsequently, the sub-feature map $x_{1}$ is fed into the offset branch, where an offset generator dynamically learns the corresponding sampling offsets $\Delta p_{l}$ and modulation masks $\Delta m_{n}$ for small projectiles. The process is formulated as follows:

(6) $$\left\{\begin{array}{l} \Delta p_{l}=\mathrm{Offset}\left(X_{1}\right)\\ \Delta m_{n}=Sigmoid\left(\mathrm{Offset}\left(X_{1}\right)\right) \end{array}\right.,$$

Due to the geometric deformation of the projectile target, the offsets need to be adaptively adjusted spatially to fit the deformed target morphology. The offsets $\Delta p_{l}$ are generally floating-point numbers, and their corresponding sampling points $p_{0}+p_{l}+\Delta p_{l}$ may not fall exactly on integer coordinates. To address this, bilinear interpolation is employed to obtain the feature response at arbitrary floating-point coordinates:

(7) $$\left\{\begin{array}{l} x\left(p\right)=\sum_{q}G\left(q,p\right)\cdot X\left(q\right)\\ G\left(q,p\right)=g\left(q_{x},p_{x}\right)\cdot g\left(q_{y},p_{y}\right) \end{array}\right.,$$

where $g\left(a,b\right)=\max\left(0,1-\left|a-b\right|\right)$ is the bilinear interpolation kernel.

After deformable convolution obtains the feature values at arbitrary floating-point coordinates through bilinear interpolation, the response of its output feature $y_{1}$ at position $p_{0}$ is given by:

(8) $$y_{1}\left(p_{0}\right)=\sum_{p_{l}\in R}w\left(p_{l}\right)\cdot x\left(p_{0}+p_{l}+\Delta p_{l}\right)\cdot \Delta m_{n},$$

Here, R denotes the regular sampling grid of the convolution kernel, and $p_{l}$ is the learned floating-point offset that allows the sampling points $p_{0}+p_{l}+\Delta p_{l}$ to align with the actual contour of the projectile. $\Delta m_{n}$ is a scalar mask learned through the Sigmoid function, with a value between 0 and 1, which dynamically suppresses the contribution of sampling points corresponding to false targets through weighting. Through the above mechanism, the convolution kernel can adaptively adjust the sampling grid according to the actual geometric shapes of projectiles and false targets. This adjustment prevents background pixels from being included in the feature extraction process. At the same time, it ensures that geometrically corrected projectile features are extracted.

Subsequently, $x_{2}$ is fed into a straight through path, where depth-wise separable convolution is employed to extract the fundamental contour features of the projectile, supplementing spatial structural constraints while preserving original details to obtain the output $y_{2}$.

The outputs $y_{1}$ and $y_{2}$ from the two branches are then subjected to cross-channel interaction and nonlinear transformation to reinforce the representation of effective projectile features. A residual connection is further introduced to enhance the feature reuse capability and training stability of the module. The final output feature map $z_{2}$ is obtained:

(9) $$z_{2}=Conv\left(Concat\left(y_{1},y_{2}\right)\right)+Conv\left(z_{1}\right),$$

### 3.4. False-Target Interference Suppression Based on Content-Guided Multi-Scale Feature Aggregation

**Figure 6:**
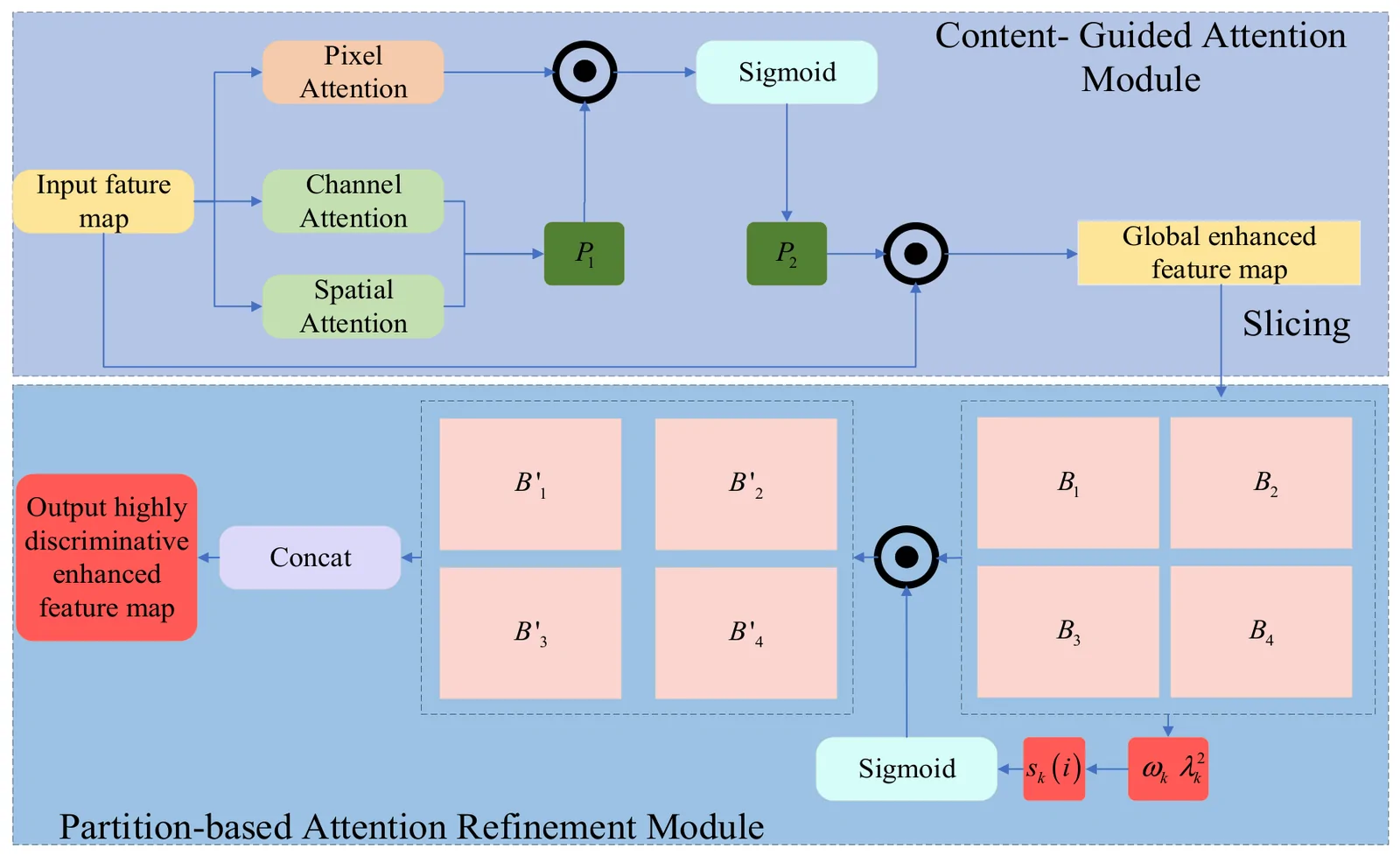
Content-guided multi-scale feature aggregation module processing flow.

The linear array CCD is deployed in an open outdoor range environment, where near-lens flying objects such as mosquitoes and sand particles are inevitably introduced during imaging. Projectiles generally exhibit relatively clear edges and a relatively uniform internal grayscale. However, near-lens flying objects have more blurred edges due to being out of focus or motion blur. There are differences between the two in terms of shape regularity, edge clarity, and gray level distribution, although the feature pyramid structure adopted by the conventional YOLOv11 neck network can achieve preliminary fusion of multi-scale features. However, the difference in the discrimination between the false target and the projectile targets is only in the extremely weak channel response and the local texture level. The cross-scale fusion method of the feature pyramid is difficult to use to explicitly model and selectively enhance such fine-grained features. To solve the problem, this study designs a Content-guided Multi-scale Feature Aggregation (CMFA) module in the neck network. Through a “channel–spatial–pixel” three-level collaborative attention mechanism, it progressively focuses on the key areas of the projectile from coarse to fine. It precisely captures the subtle discriminative differences between the projectile and the false target at the pixel-level features, thereby effectively suppressing the interference from false targets. The module flow is shown in Figure 6.

The Content-Guided Attention (CGA) unit adopts a “channel–space–pixel” three-level collaborative attention mechanism to model the input features F from coarse to fine. It performs three attention branches in parallel. The channel attention branch models the dependencies among channels and selects those that contain discriminative information, highlighting the differences between projectiles and false targets in texture, edge, and other semantic aspects. The spatial attention branch locates the spatial region where the target resides. The pixel attention branch independently evaluates the importance of each pixel position, laying the foundation for subsequent fine discrimination.

The outputs of the channel attention and spatial attention are first fused to generate a coarse-grained spatial attention map $p_{1}$, which enables the network to initially obtain the spatial location information of the projectile target. This reduces the search range from the entire image to the candidate area, effectively eliminating background interference that is far from the target. Subsequently, $p_{1}$ is combined with the output of the pixel attention branch through element-wise multiplication, followed by a Sigmoid activation function to produce the fine-grained attention weights $p_{2}$. In this process, the coarse-grained attention map $p_{1}$ shared across channels is multiplied element-wise with the channel-independent pixel attention, enabling each channel to obtain its own fine-grained attention weights, thereby achieving input-content-guided channel-specific focusing.

Through this mechanism, the channel-specific fine-grained attention can selectively strengthen the directional channel responses corresponding to the regular edges of projectiles, while suppressing the unstructured channel activations induced by false targets. Finally, $p_{2}$ is weighted element-wise with F to obtain the initially enhanced feature $F_{final}$, completing the attention refinement process from global coarse localization to channel-specific detail focusing.

Although the CGA unit achieves channel-specific fine-grained attention, it operates over the global spatial range. When the feature map size is large, the local edge details of weak and small projectiles tend to be diluted by global statistical features. Under this global averaging effect, the subtle edge regularity differences between projectiles and false targets become even more difficult to distinguish. The split-wise attention refinement module introduces a split-wise processing strategy. This strategy splits the global features into local sub-blocks, re-evaluating the edge saliency of each pixel within its local context. As a result, the continuous edges of projectiles stand out against cluttered background textures within each local block. This amplifies the discriminative differences in edge characteristics between projectiles and false targets.

The Partition-based Attention Refinement (PAR) unit first splits $F_{final}$ along the spatial dimensions into non-overlapping blocks, obtaining four local sub-blocks $\left\{B_{1},B_{2},B_{3},B_{4}\right\}$, each with a spatial size of $\left(H/2\right)\times \left(W/2\right)$. For each sub-block $B_{k}\left(k=1,2,3,4\right)$, the mean $\mu_{k}$ and variance $\lambda_{k}$ of its internal pixels are computed. The saliency score $s_{k}\left(i\right)$ for each pixel can be calculated as:

(10) $$s_{k}\left(i\right)=\frac{\left(B_{k}\left(i\right)-\omega_{k}\right)}{\lambda_{k}^{2}+\varepsilon },$$

Subsequently, the significance scores are activated through the Sigmoid function to generate intra-block attention weights. Then, these weights are applied element-wise to the initial sub-blocks $B_{k}$ to achieve local enhancement. The mathematical expression of this process is as follows:

(11) $${\hat{B}}_{k}\left(i\right)=B_{k}\left(i\right)⊙\mathrm{Sigmiod}\left(s_{k}\left(i\right)\right),$$

Here, $s_{k}\left(i\right)$ is computed based on the spatial inhibition principle of SimAM. It reflects the linear separability of each pixel within its local neighborhood. Specifically, edge pixels of the projectile exhibit continuous, oriented grayscale abruptions within the local neighborhood, forming a statistically significant difference from the surrounding background, and thus obtaining higher attention weights. In contrast, false targets with region-like blurred appearances lack such oriented continuous edges within the local neighborhood, resulting in relatively lower scores.

Through the split-wise processing, SimAM independently evaluates pixel saliency within each local sub-block, avoiding the dilution of local weak edges by global statistical features, making the edges of the projectile more easily identified and enhanced within the local context. The enhanced sub-blocks $\left\{{\hat{B}}_{1},{\hat{B}}_{2},{\hat{B}}_{3},{\hat{B}}_{4}\right\}$ are then concatenated according to their original spatial positions to restore the complete feature map, which is passed as the final output of the CMFA module to the head network.

Finally, through the two-stage progressive processing of CGA and PAR, the CMFA module effectively amplifies the discriminative differences in edge regularity between projectiles and false targets at the pixel-level feature space, providing highly discriminative feature representations for the subsequent detection head.

## 4. Experiments and Results Analysis

### 4.1. Construction of the Measurement Setup

The integrated measurement system, comprising three sky screens and a single linear array CCD, is sequentially arranged along the ballistic trajectory. The linear array CCD unit employs a Microview CDM-L2049-GM49 high-speed line-scan camera (with a resolution of 2048 × 1 pixel) equipped with a Nikon 50 mm fixed-focal-length lens, forming the image acquisition unit. The system is also equipped with a high-speed data acquisition card and a synchronous trigger, with each device installed at its designated position. Live-fire experiments were conducted using a 12.7 mm caliber gun.

The training computer used for the experiments operates on the Windows 11 operating system, with an NVIDIA GeForce RTX 4060 Laptop GPU, an i5-11400H CPU, and 16 GB of memory. The deep learning framework adopted is PyTorch 1.12.0, with CUDA version 11.3. The detailed training parameter settings are listed in Table 2.

### 4.2. Dataset Acquisition

The preparation process of the linear array CCD projectile dataset based on the three-sky-screen target and linear array CCD sensor fusion measurement system is described as follows.

To cover different illumination conditions, experiments were independently conducted within the range of 50 lx to 600 lx at intervals of 50 lx. These conditions include low-illumination (300–600 lx), medium-low-illumination (100–300 lx), and extreme-low-illumination (50 lx). Multiple independent acquisitions were performed under each condition to ensure complete illumination coverage and independence among samples.

To maintain low contrast between the target and background, imaging conditions where the grayscale of the projectile was similar to that of the background were preferentially selected during acquisition. The linear array CCD continuously captured the one-dimensional intensity sequence at a line rate of 49 kHz as the projectile passed through the field of view. The captured sequences were then stored as two-dimensional images through an image acquisition card. A total of 6352 valid projectile images were obtained, covering different flight attitudes, various low-illumination background conditions, and scenes with false-target interference.

It should be noted that although the experiment employed a 12.7 mm projectile, the uncertainties in angles during launch, together with variations in flight height as the projectile traversed the light screen, result in diverse projection morphologies and imaging scales. The pixel size of the projectile image also varies with flight height. This diversity is well reflected in the dataset composition, ensuring that the morphological and scale variations are sufficient to validate the algorithm’s recognition capability for targets with different spatial shapes and imaging scales.

Since the three-sky-screen target-integrated linear array CCD system captures images in trigger mode, the width of each triggered output image is fixed at 1024 pixels. The length, however, often reaches 3000 to 4000 pixels to ensure complete capture of the projectile trajectory across the field of view. This size is far larger than the input dimensions of conventional object detection networks, and direct input would significantly reduce network efficiency. Therefore, these images must be cropped before being fed into the network.

To enable effective cropping while preserving the target information, the bounding box and centroid pixel coordinates of the projectile target were manually annotated for each image. To ensure sample independence among the training, validation, and test sets, all original images were first divided into training, validation, and test sets at a ratio of 7:2:1. This ensures that the same original image and its cropped patches do not appear across different sets. Subsequently, sub-regions of 600 × 800 pixels were cropped from each image centered at the annotated bounding box, including typical target regions such as the projectile and near-lens flying objects. The cropped sub-regions then served as the input samples for network training and evaluation, forming the complete linear array CCD projectile dataset. Image annotation was performed independently by two researchers using the LabelImg tool, annotating the bounding boxes and centroid pixel coordinates of the projectile targets. The annotation guideline required that the bounding boxes closely fit the outer rectangle of the projectile targets, and distinguish projectiles from near-lens flying objects (the latter were not annotated). After the initial annotation, all results were reviewed image by image by a third experienced researcher, and samples with inconsistent annotations were corrected or removed.

**Figure 7:**
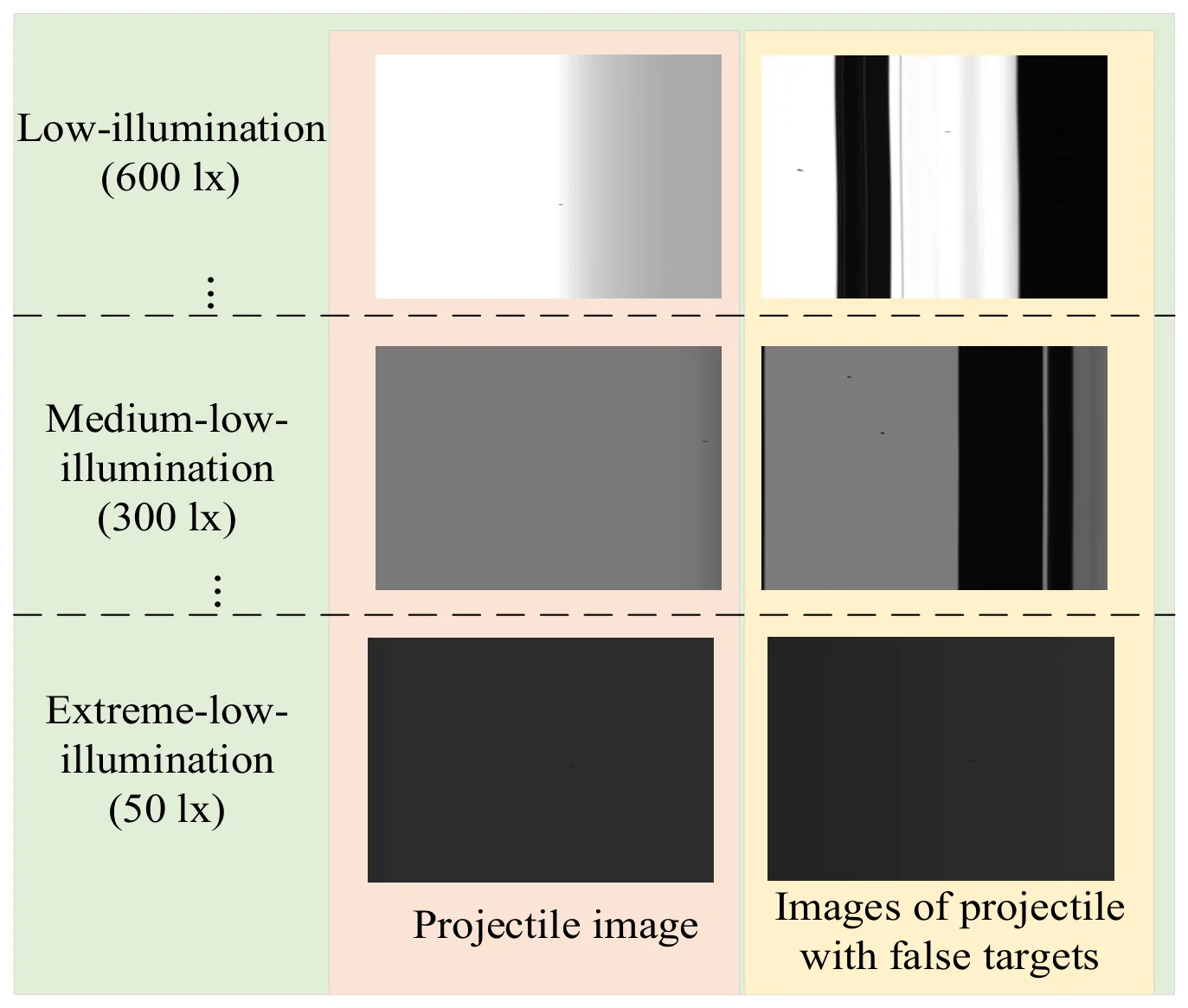
The dataset of projectiles formed through collection in different situations.

The dataset contains a total of 6352 samples, including 4446 in the training set, 1270 in the validation set, and 636 in the test set. In terms of illumination distribution, 896 samples (14.1%) were captured under low-illumination (300–600 lx), 2480 samples (39.0%) under medium-low-illumination (100–300 lx), and 2976 samples (46.9%) under extreme-low-illumination (50 lx). The low-illumination and extreme-low-illumination samples together account for 85.9% of the total, consistent with the research focus on low-illumination scenes. In terms of false-target distribution, 1432 samples (22.5%) contain near-lens flying object interference. Among these, 1002 are in the training set, 215 in the validation set, and 215 in the test set. The proportion of interference samples in each subset remains generally consistent with the overall distribution. Some examples of the dataset are shown in Figure 7.

### 4.3. Experimental Evaluation Metrics

In this experiment, Precision (P), Recall (R), False Alarm Rate (FAR), and Accuracy (AC) are adopted as the evaluation metrics for model performance. The corresponding calculation formulas are presented as follows:

(12) $$\left\{\begin{array}{l} P=\frac{TP}{TP+FP}\\ R=\frac{TP}{TP+FN}\\ FAR=\frac{FP}{FP+TN}\\ Accuracy=\frac{TP+TN}{TP+FP+TN+FN} \end{array}\right.,$$

where TP denotes the number of true targets correctly identified by the model; FP refers to the number of backgrounds or noises misclassified as targets; FN is the number of actual targets missed by the model; and TN represents the number of regions correctly judged as background. N denotes the total number of classes.

### 4.4. Validation Experiments

To verify the effectiveness of the proposed algorithm under low-illumination conditions and its adaptability to different illumination environments, experiments were conducted on the self-built linear array CCD low-illumination projectile image dataset.

**Figure 8:**
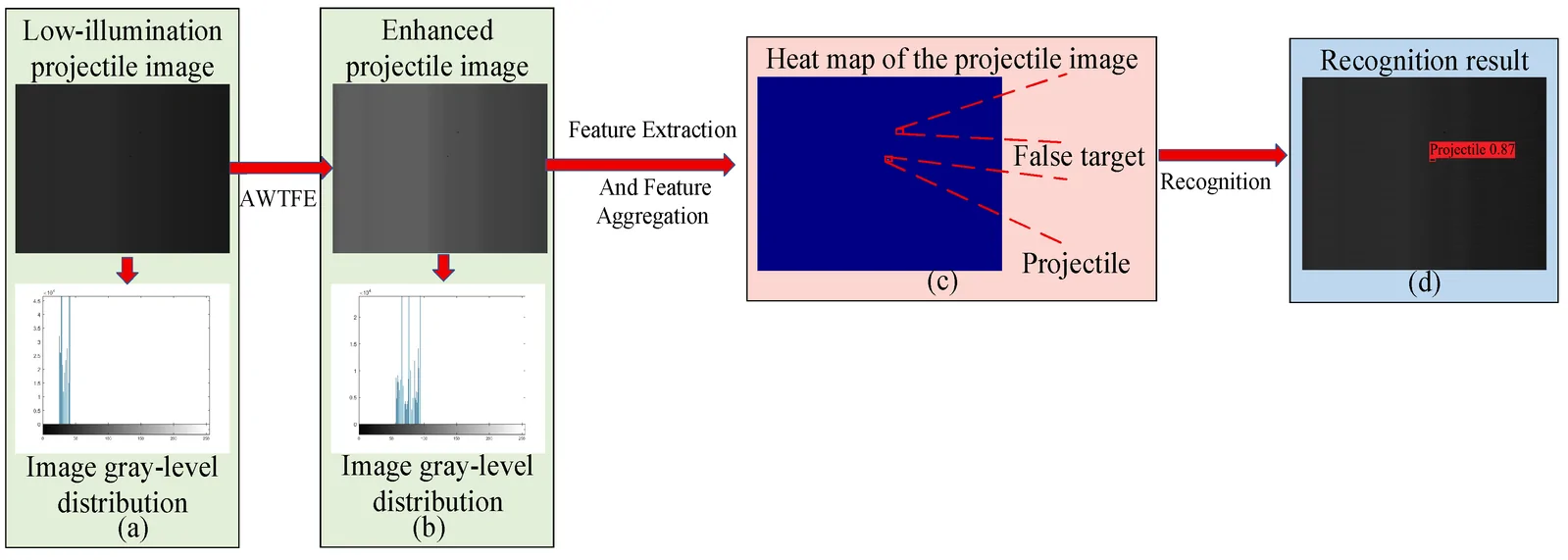
Processing and recognition of projectile images with false targets under extreme-low-illumination (50 lx) conditions. (**a**) Low-illumination projectile image; (**b**) Image of the projectile after frequency-domain enhancement; (**c**) Heat map of the projectile image; (**d**) Low-illumination projectile image recognition result.

Figure 8 shows the processing pipeline and recognition results for a projectile image containing false targets under extreme-low-illumination (50 lx) conditions. Specifically, Figure 8a shows the original captured image, where the grayscale distributions of the projectile and false targets highly overlap, making them difficult to distinguish based on visual features. Figure 8b presents the grayscale distribution after frequency-domain enhancement by the AWTFE module. It can be seen that the grayscale contrast of the projectile region is significantly restored, and the distinguishability between the target and background is notably improved. Figure 8c displays the feature heat map extracted by the FEMFF-YOLOv11 network. The area where the projectile is located exhibits a significantly higher response, while the response intensity in the false-target area is noticeably lower. These two areas form a clear distinction at the feature level. Figure 8d shows the final recognition result, where the network accurately locates the projectile position and effectively excludes false-target interference.

To further verify the recognition performance of the proposed algorithm under different illumination conditions, tests were conducted under three typical illumination levels: low-illumination (600 lx), medium-low-illumination (300 lx), and extreme-low-illumination (50 lx). The experimental results are presented in Table 3.

As shown in Table 2, the proposed algorithm demonstrates excellent recognition performance under all three illumination conditions. Under 600 lx illumination, the algorithm achieves a precision of 92.36% and a recall of 90.12%, with a false alarm rate of only 1.24%, attaining the best performance across all metrics. Under medium-low-illumination conditions of 300 lx, the precision and recall are 90.88% and 85.13%, respectively, with only a slight degradation in performance. Under extremely low illumination conditions of 50 lx, the algorithm still maintains a precision of 88.32% and a recall of 82.56%. The overall accuracy decreases from 91.85% at 600 lx to 85.91% at 50 lx, with a total drop of 5.94%. The false alarm rate remains below 2.34% across all three conditions.

**Figure 9:**
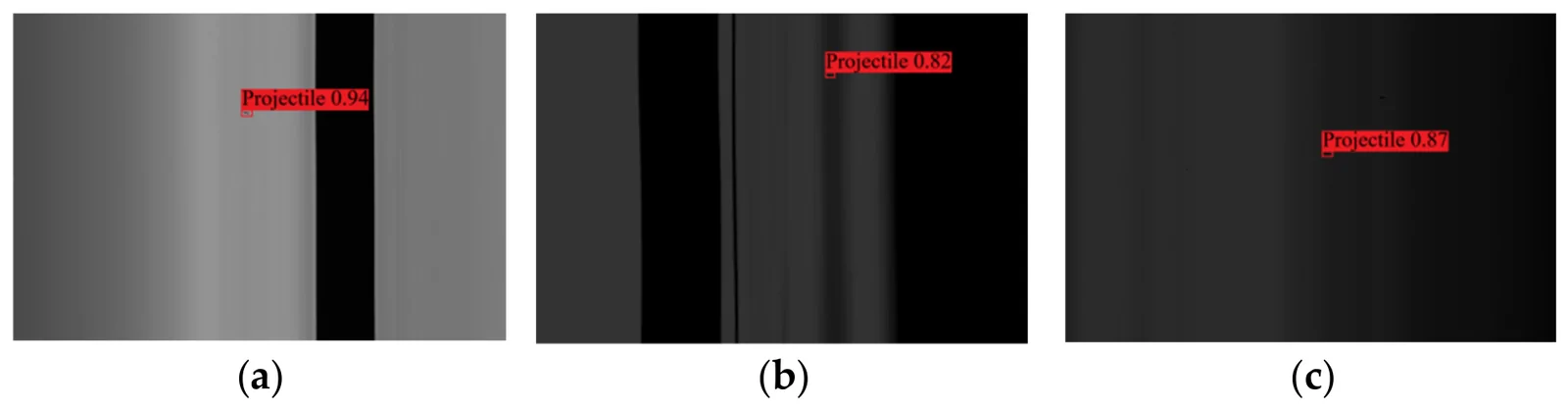
Recognition results of this algorithm in different scenes. (**a**) Recognition results of projectile images collected under low illumination(600 lx); (**b**) Recognition results of projectile images collected under medium-low-illumination(300 lx); (**c**) Recognition results of projectile images collected under extreme-low-illumination(50 lx).

Figure 9 presents a comparison of recognition results under three typical conditions. Specifically, Figure 9a shows an image acquired under low illumination, where the target is clear and the background is relatively simple, and the recognition result is accurate. Figure 9b shows an image acquired under medium-low-illumination conditions, where the contrast between the target and background is low and the edges are blurred; however, the algorithm can still effectively localize the projectile. Figure 9c shows an image acquired under extreme-low-illuminations with false-target interference, where near-lens flying objects are simultaneously present. Despite the weak target signal and similar interference, the proposed algorithm can still accurately identify the real projectile and effectively exclude false targets. The proposed algorithm achieves high recognition rates under all three conditions, verifying its robustness and effectiveness in the task of weak and small projectile target recognition under low illumination.

### 4.5. Comparative Experiments

#### 4.5.1. Projectile Recognition Performance Analysis

To verify the performance advantages of FEMFF-YOLOv11 in the task of low-illumination weak and small projectile target recognition, several mainstream object detection algorithms were selected as comparison methods and evaluated on the self-built linear array CCD low-illumination projectile image dataset. The comparison methods include the baseline model YOLOv11, the lightweight detection network YOLO-DSC, PPM-YOLOv11, which incorporates attention mechanisms, and NUDTNet designed for low-contrast scenes. These methods cover different technical approaches, including lightweight design, attention enhancement, and low-illumination optimization, and are thus highly representative. The same experimental settings and evaluation metrics were adopted to ensure fair comparison. The experimental results are presented in Table 4 and Table 5.

Under medium-low-illumination (300 lx), FEMFF-YOLOv11 achieves the best performance across all metrics, with precision of 90.88%, recall of 85.13%, FAR of 1.86%, and accuracy of 87.35%, while maintaining the smallest standard deviations, typically below 0.80%. Compared with YOLOv11, our method improves accuracy by 7.49%, recall by 4.66%, and reduces FAR by 3.25%. Compared with NUDTNet, PPM-YOLOv11, and YOLO-DSC, our method improves accuracy by 4.88%, 3.23%, and 3.72%, respectively. It is worth noting that although NUDTNet achieves relatively high precision (88.36%) at 300 lx due to its low-contrast-oriented design, its accuracy (82.47%) lags behind that of PPM-YOLOv11 and YOLO-DSC because of its lower recall (81.54%), indicating that NUDTNet suffers from obvious missed detection problems under low-illumination conditions. In terms of inference speed, YOLO-DSC achieves the fastest time of 2.8 ± 0.2 ms due to its lightweight architecture, followed by YOLOv11 at 4.2 ± 0.3 ms. Our method ranks third at 6.5 ± 0.4 ms, a moderate increase over YOLOv11, while PPM-YOLOv11 and NUDTNet require 8.7 ± 0.6 ms and 12.3 ± 1.0 ms, respectively. The moderate increase in inference time, attributable to the additional AWTFE, DOC, and CMFA modules, is well justified by the substantial gains in precision, recall, and FAR suppression.

As shown in Table 5, when illuminance drops to 50 lx, all algorithms deteriorate significantly. NUDTNet’s accuracy drops from 82.47% to 75.23%, with its high standard deviation, indicating severe instability under extreme-low-illumination. YOLO-DSC exhibits the most pronounced degradation in precision (from 83.58% to 70.36%) and FAR (from 6.95% to 9.12%), confirming that its lightweight design struggles to maintain reliable feature extraction when the illumination becomes extremely low. PPM-YOLOv11 also shows notable performance loss, with its FAR nearly doubling from 2.97% to 5.86%, suggesting that its attention mechanism tends to amplify noise under extreme conditions. In contrast, FEMFF-YOLOv11 maintains accuracy at 85.91%, only 1.44% lower than at 300 lx, while preserving precision of 88.32% and recall of 82.56%. The standard deviations of our method remain consistently low. This confirms that our method maintains robust stability under extreme-low-illumination. Since inference time is architecture-dependent and independent of input content, the inference times remain identical to those in Table 4.

**Figure 10:**
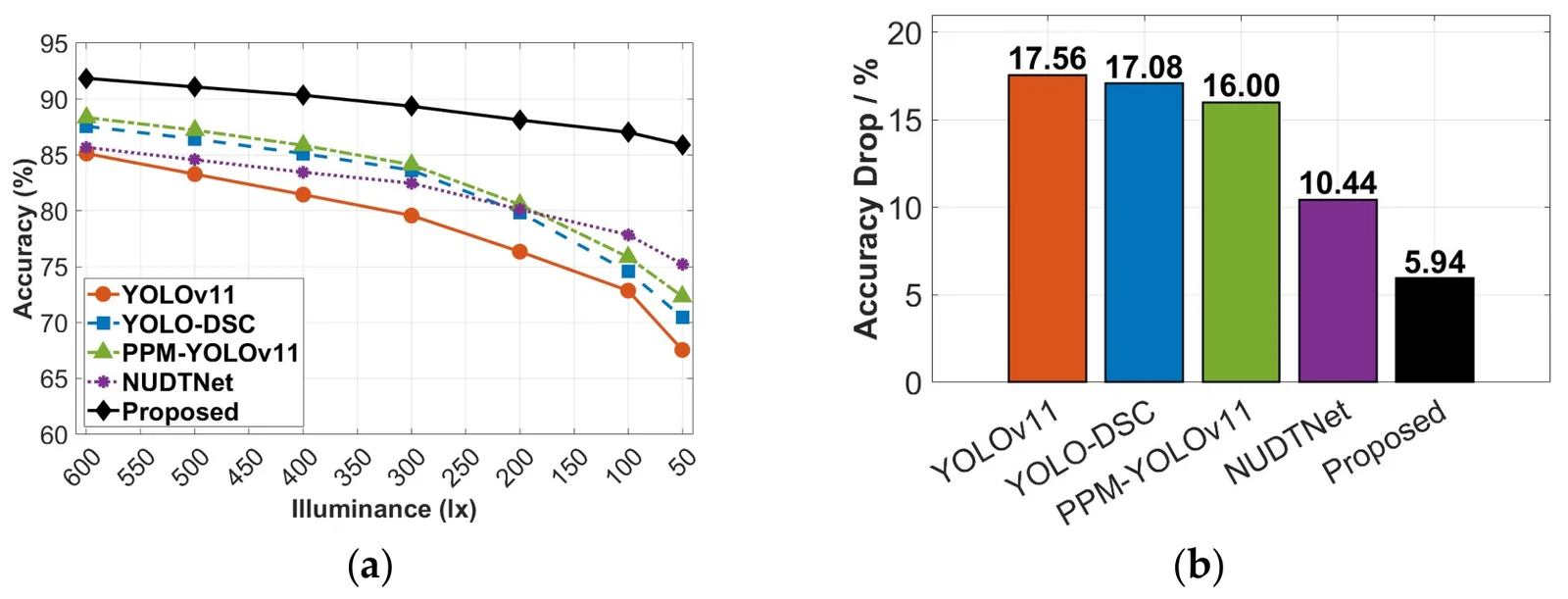
Performance evaluation of different algorithms under varying illumination conditions: (**a**) accuracy versus illuminance curves; (**b**) accuracy drop from 600 lx to 50 lx.

To further evaluate the performance of all algorithms under varying illumination conditions, independent experiments were conducted across a range of illuminance levels. All algorithms were trained on the same training set and then independently tested on the same test set under each illuminance condition, to eliminate the influence of training data distribution discrepancies on the evaluation results. The illuminance range covers 50 lx to 600 lx (including low-illumination, medium-low-illumination, and extreme-low-illumination), with a sampling interval of 50 lx. The recognition accuracy of each algorithm was tested at each illumination level, and the relationship curve between the accuracy and the illumination was plotted. The changes in accuracy were also shown, as depicted in Figure 10. This provides a clear and intuitive illustration of the performance trends and robustness of different algorithms under different lighting conditions.

As shown in Figure 10a, the accuracy of all algorithms decreases as illumination drops, but the degradation patterns differ substantially. At 600 lx, the proposed method achieves an accuracy of 91.85%, while YOLOv11, YOLO-DSC, PPM-YOLOv11, and NUDTNet achieve 85.12%, 87.56%, 88.34%, and 85.67%, respectively. At 300 lx, the accuracies drop to 79.58%, 83.63%, 84.12%, 82.47%, and 89.35%, with the proposed method still maintaining the highest performance. Under extreme-low-illumination (50 lx), YOLOv11, YOLO-DSC, PPM-YOLOv11, and NUDTNet drop to 67.56%, 70.48%, 72.34%, and 75.23%, while the proposed method remains at 85.91%, further widening its advantage. Figure 10b further quantifies the accuracy drop from 600 lx to 50 lx for each algorithm. The proposed method shows the smallest degradation (5.94%), showing the most gradual decline among the compared methods and benefiting from its low-contrast oriented design. These results consistently confirm the strong robustness and adaptability of the proposed method to illumination change.

#### 4.5.2. Visualization Analysis of Recognition Results

**Figure 11:**
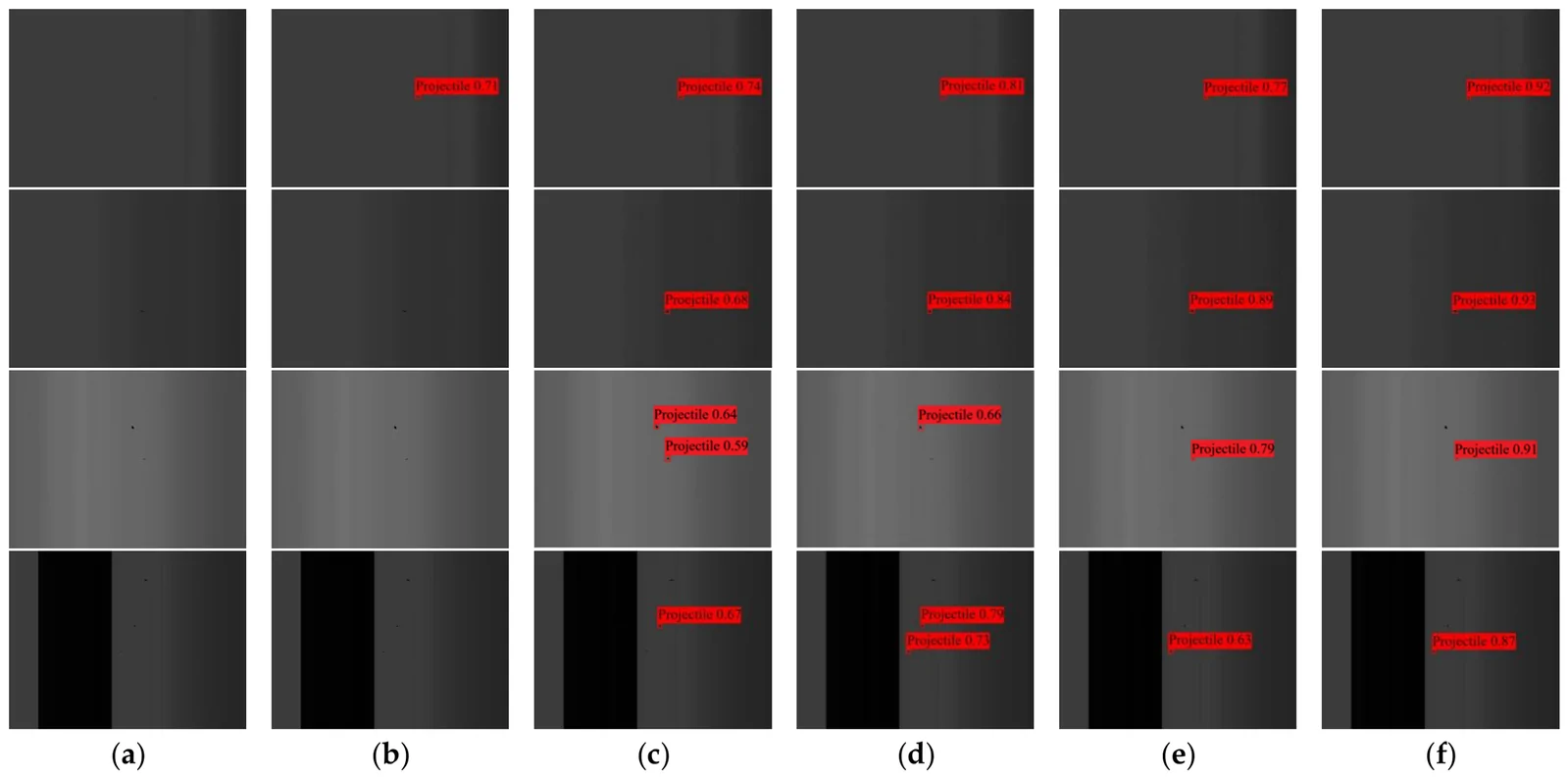
Visualization of recognition results under different conditions: (**a**) original images; (**b**) YOLOv11; (**c**) YOLO-DSC; (**d**) PPM-YOLOv11; (**e**) NUDTNet; (**f**) proposed algorithm.

To validate the recognition performance of the improved algorithm, real-scene low-illumination projectile images captured by the linear array CCD are employed for testing. The recognition performance of the compared algorithms is evaluated through visualization analysis across different scenes. Specifically, tests are conducted on four typical scenes, covering cases of low image illumination and the presence of false targets, to comprehensively assess the effectiveness of the proposed algorithm. Figure 11 compares recognition results under four typical low-illumination conditions.

Under low illumination without interference, all methods detect the target, but our method gives the highest confidence. Under low illumination with a complex background, YOLOv11 fails due to vanishing CIoU gradients, YOLO-DSC gives low confidence due to its lightweight design, PPM-YOLOv11 provides limited enhancement, and NUDTNet loses local details. Our method maintains the highest confidence and best localization. Under low illumination with false targets, YOLOv11 and YOLO-DSC fail or misclassify, while PPM-YOLOv11 and NUDTNet show low confidence. Our method distinguishes real targets from false ones with the highest confidence. Under combined interference, only our method accurately identifies the target without missed detections or false positives.

These results demonstrate the effectiveness and superiority of FEMFF-YOLOv11 in complex low-illumination scenes.

### 4.6. Ablation Experiments

To verify the effectiveness and necessity of each core innovative module proposed in this study, ablation experiments are conducted on the self-built low-illumination linear array CCD projectile image dataset under all illumination conditions. YOLOv11 is adopted as the baseline model, and modules are successively added into the network. The performance changes of the projectile recognition algorithm before and after adding each module are compared through the variations in evaluation metrics, thereby demonstrating the effectiveness of the proposed improvements. Table 6 presents the performance changes of the projectile recognition algorithm with the sequential addition of modules.

As shown in Table 6, single-module analysis reveals distinct performance improvements. AWTFE increases recall by 8.36%, reduces false alarm rate from 5.11% to 2.86%, but improves precision by only 2.78%, because it recovers weak edges through frequency-domain high-frequency enhancement, acting as signal restoration rather than feature selection, thus significantly improving the detection rate while limitedly raising precision. C3k2-DOC achieves the largest precision gain from 79.58% to 85.73%, as deformable convolution adapts to irregular projectile shapes via dynamic offsets, enhancing geometric feature extraction and boosting confidence in true positives. CMFA raises recall to 84.26%, and lowers the false alarm rate to 3.42%, but precision improves by only 2.07%, as its attention mechanism distinguishes true and false targets but its discriminative ability is constrained by the input signal-to-noise ratio, relying on AWTFE front-end enhancement.

To further investigate the effects among modules, dual-module combinations are evaluated. AWTFE with C3k2-DOC achieves the best dual-module accuracy of 89.20%, improving 3.40% over AWTFE alone and 5.08% over C3k2-DOC alone, indicating that high-quality feature maps provide clearer edge guidance for deformable offset convolution, forming effective synergy in feature extraction. AWTFE with CMFA achieves the highest recall of 90.24%, a 3.46% increase over AWTFE alone, showing that frequency-domain enhancement offers more accurate focus for attention. C3k2-DOC with CMFA attains a precision of 89.18% and an accuracy of 88.43%, demonstrating that CMFA further refines true target responses and suppresses false targets based on stable morphological features.

The full model integrates all three modules and achieves optimal metrics: precision of 90.88%, recall of 92.87%, false alarm rate of 1.25%, and overall accuracy of 92.35%. The step-by-step comparative results of the ablation experiments show that the three modules progressively advance from time-frequency domain restoration and geometric adaptation to multi-scale aggregation, with performance consistently improving at each stage, thereby validating the rationality of the design and the effectiveness of the module selection.

Recent studies have highlighted the importance of systematically evaluating model performance under varying experimental conditions, such as different random seeds and data splits [39,40]. These works demonstrate that deep learning models can exhibit sensitivity to such factors, and that rigorous multi-run evaluations are essential for establishing reliable performance benchmarks. Inspired by these methodologies, we conducted supplementary experiments to assess the stability of our method: under a fixed data split (7:2:1), the complete model was trained three times with different random seeds (42, 123, and 456) and evaluated on the same test set. The sensitivity results under different conditions are shown in Table 7.

The results show that the mean accuracy is 91.83% with a standard deviation of 0.18% under 600 lx, 87.32% with a standard deviation of 0.15% under 300 lx, and 85.88% with a standard deviation of 0.12% under 50 lx. It indicates that under all lighting conditions, the performance changes caused by random weight initialization are relatively small. In addition, with a fixed random seed (42), three different data split ratios (6:2:2, 7:1.5:1.5, and 8:1:1) were adopted to re-partition the dataset and retrain the model. The test set accuracies are 87.21%, 87.35%, and 87.08%, respectively, with a variation of less than 0.4%, confirming that the proposed method is insensitive to specific data partition settings. These supplementary experiments confirm that the performance metrics reported under the fixed random seed and 7:2:1 split ratio are representative of the method’s typical performance, addressing the methodological concerns raised in the referenced studies.

## 5. Conclusions

To address the challenges of low contrast, blurred edges, and ineffective recognition of projectile targets under low-illumination conditions in the three-sky-screen integrated linear array CCD testing system, this paper proposes FEMFF-YOLOv11, a projectile target recognition method based on frequency-domain enhancement and multi-scale feature fusion. The method integrates four key improvements: AWTFE for frequency-domain feature enhancement, DOC for geometric deformation adaptation, CMFA for false-target suppression, and a P2 detection layer for preserving the spatial resolution of weak and small targets.

Experimental results on the self-built low-illumination linear array CCD projectile dataset demonstrate that FEMFF-YOLOv11 achieves 87.35% accuracy and 85.13% recall under conventional low-illumination conditions, improving by 7.49% and 4.66% over the baseline YOLOv11. Under extreme-low-illumination (50 lx), it maintains 85.91% accuracy and 82.56% recall, significantly outperforming existing mainstream small-target detection methods. These results validate the effectiveness and robustness of the proposed method in low-illumination projectile recognition tasks. They provide reliable recognition support for the linear array CCD sensor within the three-sky-screen integrated projectile testing system.

## Figures and Tables

**Table 1:** Analysis of Images under Different Conditions.

Scene	Illumination Condition	Image Feature Description	Projectile Recognition Challenge Analysis
Figure 2a low illumination	600 lx	Clear edges, good contrast	Fewer than 8 pixels, limit spatial features
Figure 2b medium-low illumination without interference	300 lx	Blurred edges, low contrast	Grayscale close to with background
Figure 2c medium-low illumination with background submerged	300 lx	Edges submerged by backgrounds	Target coupled with background texture differences
Figure 2d extreme-low illumination with false targets	50 lx	Projectile and false targets both blurred	Low light compresses shape and edge

**Table 2:** Training parameter settings.

Training Parameter	Value
Initial learning rate	0.01
Warm-up epochs	20
Final learning rate	0.01
Training epochs	200
Optimizer	SGD
Number of images per batch	32

**Table 3:** Comparison results of recognition algorithms under different illumination conditions.

Illuminance/lx	Precision/%	Recall/%	False Alarm Rate/%	Accuracy/%
600	92.36 ± 0.52	90.12 ± 0.63	1.24 ± 0.58	91.85 ± 0.55
300	90.88 ± 0.67	85.13 ± 0.79	1.86 ± 0.72	88.35 ± 0.71
50	88.32 ± 0.84	82.56 ± 0.96	2.34 ± 0.81	85.91 ± 0.75

**Table 4:** Comparison of Different Recognition Algorithms Under Medium-Low-Illumination Conditions (300 lx).

Algorithm	Precision/%	Recall/%	FAR/%	Accuracy/%	Inference Time/ms
YOLOV11 [35]	79.58 ± 1.42	78.42 ± 1.57	5.11 ± 1.08	79.58 ± 1.35	4.2 ± 0.3
YOLO-DSC [36]	83.58 ± 1.35	80.47 ± 1.43	6.95 ± 0.95	83.63 ± 1.24	2.8 ± 0.2
PPM-YOLOv11 [37]	85.73 ± 1.08	81.89 ± 1.26	2.97 ± 0.87	84.12 ± 1.15	8.7 ± 0.6
NUDTNet [38]	88.36 ± 0.96	81.54 ± 1.33	3.72 ± 0.91	82.47 ± 1.18	12.3 ± 1.1
Proposed method	90.88 ± 0.67	85.13 ± 0.79	1.86 ± 0.72	87.35 ± 0.64	6.5 ± 0.4

**Table 5:** Comparison Of Different Recognition Algorithms Under Extreme-Low-Illumination Conditions (50 lx).

Algorithm	Precision/%	Recall/%	FAR/%	Accuracy/%	Inference Time/ms
YOLOV11	69.58 ± 2.34	65.42 ± 2.51	8.13 ± 1.26	67.56 ± 2.43	4.2 ± 0.3
YOLO-DSC	70.36 ± 2.15	68.17 ± 2.35	9.12 ± 1.35	70.48 ± 2.21	2.8 ± 0.2
PPM-YOLOv11	74.21 ± 1.96	70.53 ± 2.18	5.86 ± 1.05	72.34 ± 2.07	8.7 ± 0.6
NUDTNet	79.45 ± 1.73	73.22 ± 1.94	4.97 ± 0.98	75.23 ± 1.86	12.3 ± 1.1
Proposed method	88.32 ± 0.84	82.56 ± 0.96	2.34 ± 0.81	85.91 ± 0.75	6.5 ± 0.4

**Table 6:** Ablation experiment results (√: module included; ×: module excluded).

NO	Baseline Model	AWTFE	C3k2-DOC	CMFA	Evaluation Metric			
					Precision/%	Recall/%	FAR/%	Accuracy/%
1	YOLOv11	×	×	×	79.58 ± 1.42	78.42 ± 1.57	5.11 ± 1.08	78.50 ± 1.35
2	YOLOv11	√	×	×	82.36 ± 1.23	86.78 ± 0.85	2.86 ± 0.94	85.80 ± 0.85
3	YOLOv11	×	√	×	85.73 ± 1.08	81.89 ± 1.26	2.97 ± 0.87	84.12 ± 1.15
4	YOLOv11	×	×	√	81.65 ± 1.34	84.26 ± 0.96	3.42 ± 0.82	83.59 ± 1.08
5	YOLOv11	√	√	×	88.92 ± 0.79	89.13 ± 0.74	2.12 ± 0.78	89.20 ± 0.80
6	YOLOv11	√	×	√	85.64 ± 0.91	90.24 ± 0.68	2.56 ± 0.71	88.91 ± 0.76
7	YOLOv11	×	√	√	89.18 ± 0.73	87.62 ± 0.83	2.41 ± 0.73	88.43 ± 0.79
8	YOLOv11	√	√	√	90.88 ± 0.67	92.87 ± 0.61	1.25 ± 0.67	92.35 ± 0.64

**Table 7:** Sensitivity analysis of the proposed method to random factors.

Illuminance	Seed = 42	Seed = 123	Seed = 456	Mean ± Std (%)
600 lx	91.85	91.78	91.86	91.83 ± 0.18
300 lx	87.35	87.25	87.36	87.32 ± 0.15
50 lx	85.91	85.82	85.91	85.88 ± 0.12

## Data Availability

The data presented in this study are available upon request from the corresponding author.
